# Standing together at the helm – how employees experience employee-driven innovation in primary care

**DOI:** 10.1186/s12913-024-11090-0

**Published:** 2024-05-22

**Authors:** Sarah Samuelson, Sandra Pennbrant, Ann Svensson, Irene Svenningsson

**Affiliations:** 1 Region Västra Götaland, Research, Education, Development & Innovation (REDI), Primary Health Care, Sweden; 2 University West, Department of Health Sciences, Trollhättan, 461 86 Sweden; 3 University West, School of Business, Economics and IT, Trollhättan, 461 86 Sweden; 4 General Practice, Family Medicine, School of Public Health and Community Medicine, Institute of Medicine, Sahlgrenska Academy, University of Gothenburg, Gothenburg, Sweden

**Keywords:** Employee-Driven Innovation, Lean philosophy, Learning organisation, Primary Care, Sociocultural perspective, Qualitative Research

## Abstract

Primary care needs to find strategies to deal with today’s societal challenges and continue to deliver efficient and high-quality care. Employee-driven innovation is increasingly gaining ground as an accessible pathway to developing successful and sustainable organisations. This type of innovation is characterised by employees being engaged in the innovation process, based on a bottom-up approach. This qualitative study explores employees’ experiences of employee-driven innovation at a primary care centre in Sweden. Data are collected by focus group interviews and analysed by inductive qualitative content analysis. The result is presented with the overarching theme “Standing together at the helm” followed by three categories: “Motivating factors for practising employee-driven innovation”, “Challenges in practising employee-driven innovation” and “Benefits of employee-driven innovation”, including nine subcategories. The study found that employee-driven innovation fosters organisational innovation, empowers employees, and enhances adaptability at personal and organisational levels. This enables individual and collective learning, and facilitates the shaping, development, and adaptation of working methods to meet internal and external requirements. However, new employees encountered difficulty grasping the concept of employee-driven innovation and recognising its long-term advantages. Additionally, the demanding and task-focused environment within primary care posed challenges in sustaining efforts in innovation work. The employees also experienced a lack of external support to drive and implement some innovative ideas.

**Supplementary Information:**

The online version contains supplementary material available at 10.1186/s12913-024-11090-0.

## Introduction

Primary care, as well as healthcare in general, is highly dependent on its workforce [1] and it is argued that it is vital to strengthen employees’ innovative power to better handle societal challenges and deliver efficient and high-quality care [2]. Primary care has the potential and capability to improve health outcomes across socioeconomic levels and improve health system efficiency [3, 4]. Thus, many countries, including Sweden, have a policy agenda to strengthen, develop and transform primary care to address societal challenges such as an ageing population with an increased burden of chronic disease and heightened healthcare costs [5, 6]. In the transformation of primary care, innovations have become imperative [2]. Although often overlooked in the past, contemporary organisations are increasingly recognising employees’ potential to contribute to innovation at the workplace, such as in employee-driven innovation [7, 8]. Employees’ professional, context-specific and practice-based knowledge allows them to capture ideas, solutions to problems and suggestions for improvement that arise in daily work [7, 9], which contributes to organisational development [7] and improved performance [10]. Innovation at work is vital to ensure long-term viability and adaptability of organisations, especially in the face of ever-changing work requirements and evolving client needs [11]. Dzau and colleagues [12] stress that innovation must be actively promoted in healthcare settings by teaching, supporting, and implementing innovation in order to drive healthcare transformation.

Like many other countries, Sweden has a growing shortage of healthcare professionals [13, 14] and primary care as well as healthcare in general, struggles to recruit and retain competent healthcare professionals. These struggles tend to be even more pronounced at primary care centres in rural and sparsely populated areas. The primary care centre in this present study started to engage in employee-driven innovation after facing a challenging situation when several of the workplace’s more experienced employees left their jobs. As a result of loss of workforce and competence, work routines began to fail which in turn led to an increased workload for remaining staff, as well as difficulties in recruiting and retaining new employees due to a demanding work environment. In order to reverse this negative trend and create an attractive workplace, a continuous and structured employee-driven innovation work based on Lean thinking [15] was initiated at the primary care centre in 2016 and is still ongoing.

The research field of employee-driven innovation is steadily growing. However, in previous research most empirical studies have been conducted in the private sector, rather than the public sector (see [16–18]), which a recent literature review on employee-driven digital innovation also concludes [19]. To the best of our knowledge, no studies to date have investigated experiences of employee-driven innovation in public primary care from the employees´ perspective. To understand a phenomenon in depth, it needs to be investigated in the context in which it occurs [20]. Thus, the aim of this study is to explore employees’ experiences of employee-driven innovation in a primary care context.

### Theoretical background

#### Employee-driven innovation

Innovation can be understood as a multi-step process through which organisations transform their ideas into new or improved products, services or processes, enabling them to advance, compete and differentiate themselves in their sector [21]. The concept of innovation includes both incremental innovations, such as gradual improvements of existing practice that lead to organisational development, as well as radical innovations that lead to drastic and fundamental changes and improvements in some defined area [22]. Employee-driven innovation is a broad umbrella concept that approaches innovation from a grassroots perspective. Høyrup ([23], p.8) refers to this type of innovation as “the generation and implementation of new ideas, products, and processes – including the everyday remaking of jobs and organisational practices – originating from the interaction of employees, who are not assigned to this task”. These processes, which can be initiated, supported or even driven by employees, can be integrated into an organisation’s collaborative and management work. As this definition suggests, employee-driven innovation is based on the idea that employees at all organisational levels have potential for innovation [8, 24]. Even if employees are not formally tasked with innovating, they can contribute potentially valuable ideas as they see things in their everyday work. Thus, employees become the very driving force for innovation [8] and strategic organisational development [22], unlike research, technology, user or market-driven innovation [8]. This denotes an inclusive and democratic view of who can contribute to innovation within an organisation. However, the employee’s role and level of engagement in the innovation process may vary and Høyrup [23] suggests a typology in which employee-driven innovation processes are expressed in three generic orders. First-order employee-driven innovation is described as a pure bottom-up process where employees actively shape and refine their practices throughout the workday. These actions involve making subtle adjustments or improvements to enhance outcomes and workflow, all without the explicit aim of innovation and without direct managerial intervention. Second-order employee-driven innovation is a combination of bottom-up and top-down processes, where innovation processes are initiated by employees, but structured and formalised by management. Third-order employee-driven innovation denotes a top-down process where managers invite employees to participate in already-planned innovation activities. Common to all employee-driven processes, however, is that they cannot be separated from social processes in the organisation but are embedded in the daily work activities of job enactment [23], which puts a focus on systems for innovation grounded in daily practice that become a part of an organisation’s culture [12].

#### Employee-driven innovation from a sociocultural perspective

The theoretical starting point in this study is the sociocultural perspective [25, 26], where the focus is on individuals being in social contexts where different activities and interactions are shaped by the historical, cultural, social and institutional context. These human activities are mediated by various cultural tools, such as artefacts, symbols, language or systems of knowledge. Cultural tools play a vital role in the co-construction of knowledge as they mediate between people and their social context [27, 28]. It is through communication, with the help of cultural tools, that people learn new ways of thinking, reasoning and behaving. The relationship between thinking, communication and action is situational, and the primary focus is to create understanding and meaning between the context and the different activities [25, 26]. In the sociocultural perspective, the Zone of Proximal Development (ZPD) is a central concept in comprehending how individuals learn through social interaction and collaborating with others. It represents the gap between what learners can accomplish independently based on their current abilities and what they can achieve with guidance or through collaboration with more capable peers [26].

Therefore, as a phenomenon, learning is situated in a sociocultural context [26, 29, 30] such as workplaces [31]. Innovation at work and learning are closely intertwined and consist of interdependent processes of change [11, 32] and therefore learning plays a key role in employee-driven innovation. Learning and knowledge creation processes at work define new problems and challenges that drive the development of new knowledge required to solve them [33].

The primary care sector is an organised institutional system of socially established activities [28]. How people that are engaged in employee-driven innovation create understanding may depend on the organisation’s knowledge culture and tradition, that is, the organisational conditions that prevail. Vygotsky [26] believes that understanding is created in interaction with others. This means that different activities and learning depend on how the participants have understood them. Thus, collaboration and learning does not take place in a social vacuum, but in a sociocultural context with cultural, historical and social factors together with those involved [25, 26]. Primary care can be seen as an institution with its own values, ideas and knowledge. How employees’ experience employee-driven innovation can be understood based on the employees’ previous social and cultural experiences of organisational development and their understanding of the concept of innovation.

## Method

### Study design

This is a qualitative exploratory study with an insider perspective. An insider perspective implies that the researcher is a member of the organisation, group or community being studied [34, 35]. In this study, the first author workes as a district nurse at the primary care centre and has been engaged in employee-driven innovation efforts since their inception in 2016.

### Study context

The study was conducted at a primary care centre in a rural municipality on the west cost of Sweden.

### Outline of the employee-driven innovation work

**Fig. 1:**
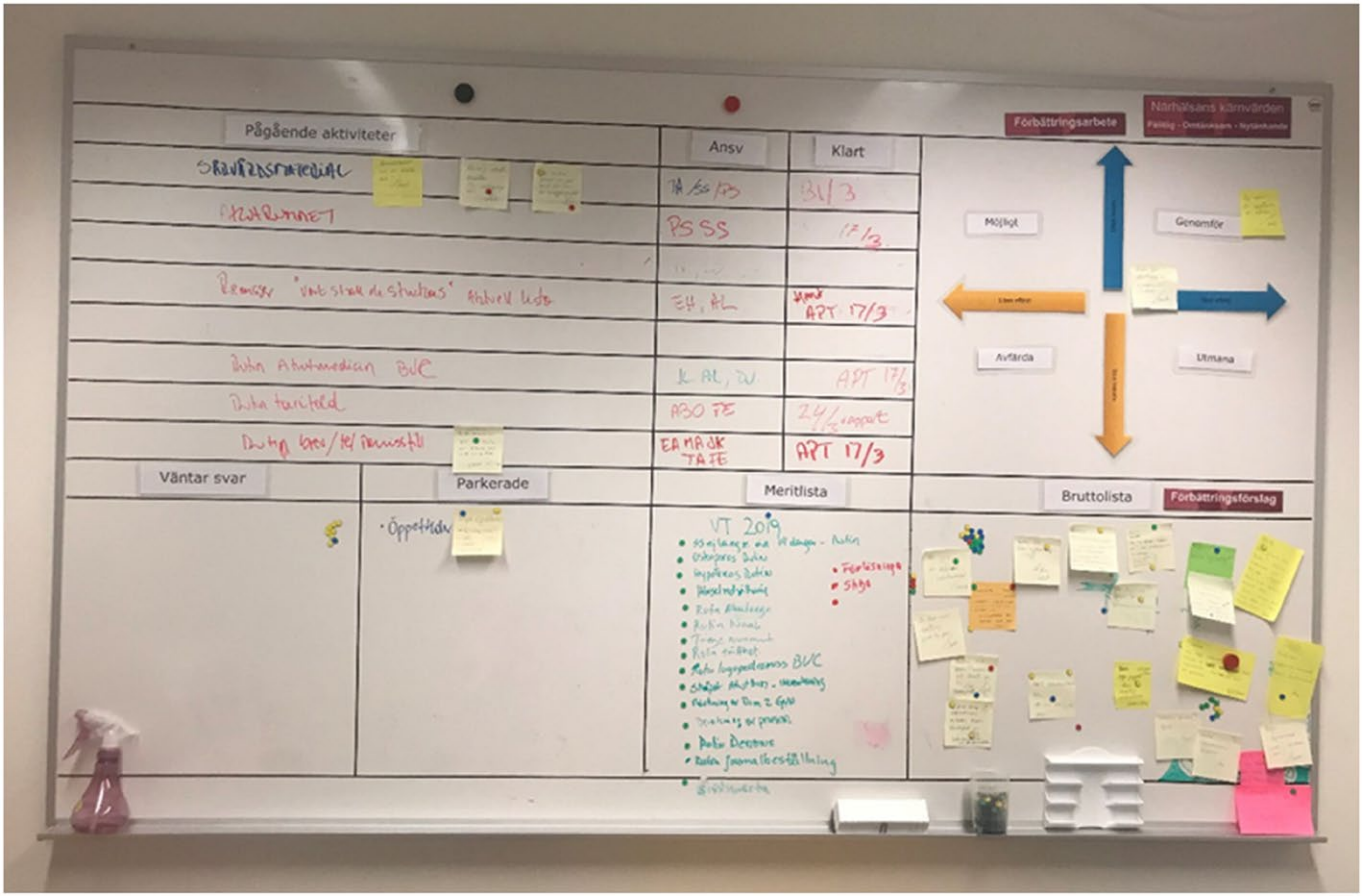
Lean board

This study explores employees´ experience of what Høyrup [23] terms as second-order employee-driven innovation, a process that involves a combination of bottom-up and top-down approaches. In the employee-driven innovation process, employees are responsible for idea generation, problem solving and implementation, while the manager is tasked with establishing and maintaining collaboration structures, allocating necessary resources (such as time and education), and offering support if needed. The framework of Lean thinking [15] forms the very basis of the employee-driven innovation work at the primary care centre and a Lean board is used as a visual tool [36] in the innovation process. Lean thinking includes both socio-technical and operational aspects of Lean [15], emphasising the employees´ role in the Lean process along with the tool used. In healthcare settings, Lean boards may be used as a project management system, to structure improvements and innovation efforts. Furthermore, the tool can foster and empower employees to engage in the innovation process [37, 38]. At the primary care centre, the Lean board helps the manager to organise and visualise the innovation process. Tasks that are upcoming, in progress and completed are displayed and catagorised in different columns on the Lean board. In this case, the main columns were arranged into the following categories: suggested improvements, ongoing activities, answerable, time schedule, and completed (Fig. 1).

**Fig. 2:**
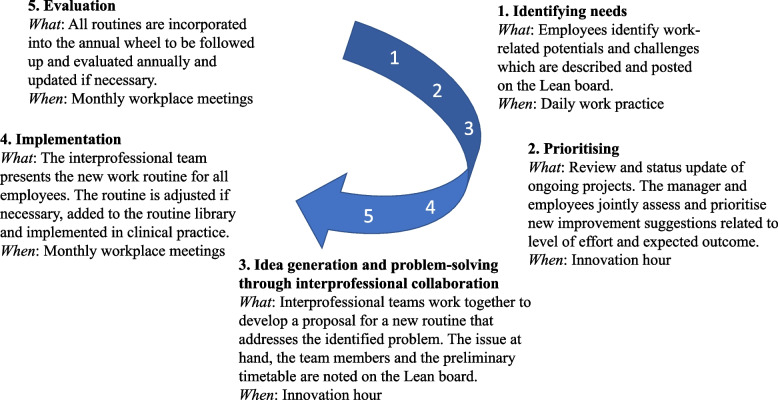
The employee-driven innovation process

Thus, at the primary care centre employee-driven innovation is practised in the following way (Fig. 2): (1) Employees identify problems, ideas, or improvement suggestions in their daily work and describe these on ‘improvement notes’ that are then displayed on the Lean board. (2) Every Tuesday one hour is scheduled for joint innovation work. Initially, everyone convenes around the Lean board where the manager provides a succinct overview of ongoing projects and receives status updates. Then the manager and the employees jointly discuss and assess new improvement notes on the board and, upon consensus, new projects are initiated. Following this (3), employees collaborate in interprofessional teams, whether working on new or existing projects, to devise innovative work routines aimed at addressing identified issues. (4) When completed, the routine is presented to all employees at a monthly workplace meeting for final assessment and adjustment. Thereafter, the routine is included in the primary care centre’s joint digital routine library which is accessible to all employees. (5) Finally, all routines are evaluated annually and updated if necessary.

### Sampling and participants

To ensure a rich description of the phenomenon being studied, the sampling was purposive [20]. The primary care centre in the study was selected because employee-driven innovation is incorporated into weekly practice. At the time of the study the primary care centre had 23 employees excluding the manager and the first author. To be included in the study, the employee needed to have participated in employee-driven innovation activities. Employees with managerial/team leader positions were excluded. In total, five employees were excluded, three (one podiatrist, one general practitioner and one psychologist) due to not participating in the innovation work because of working part-time, one nurse due to managerial position and one resident physician was not asked to participate because at the time of the focus group interview, he was stationed elsewhere. After receiving approval from the manager of the primary care centre, all employees who met the inclusion criteria received written (e-mail) and oral information about the aim, procedure, and ethical aspects of the study. All agreed to participate and confirmed this by completing a consent form. Next, all participants were divided into three interprofessional focus groups with five to seven employees in each group (Table 1). However, two employees (one medical secretary and one caretaker) dropped out due to illness at the time of the third focus group interview and therefore there were only three participants in that group.

**Table 1:** Focus group participants

Focus Group 1 (n:6)	Focus Group 2 (n:7)	Focus Group 3 (n:3)
2 nurses/district nurses	3 nurses/district nurses	2 nurses/district nurses
1 nurse assistant	2 medical secretaries	1 psychotherapist
1 medical secretary	1 resident physician	
2 resident physicians	1 general practitioner	

### Data collection

Data were collected by focus group interviews. Focus groups are of benefit when the goal is to understand how individuals collectively construct meaning through social interactions [20, 39]. Given that innovation is largely a socially constructed process [23, 40], we found focus group interviews to be a suitable method for data collection. Three focus group interviews, lasting on average 59 min (49–70), were conducted face-to-face at the primary care centre at scheduled times during May 2021. The first (SS) and the last author (IS) conducted the focus group interviews together and took turns to act as moderator and note-taking observer at the different focus group interviews. The moderator used a semi-structured interview guide that contained open-ended questions on the subject area of employee-driven innovation in a primary care context, for example, how the innovation work started, how and why they innovate in daily practice and what are the benefits/challenges with employee-driven innovation (See Supplementary File 1). Probing questions, such as “why is that?” and “can you tell us more about your thoughts on…?”, were asked to encourage in-depth reflections on the issue discussed [41].

### Analysis

Data were analysed by inductive content analysis with a focus on both the manifest and the latent content of the interview texts [42, 43], performed in the following six steps. First, all recorded material was transcribed verbatim. Second, the first and the last author initially read all the interview texts several times to get a sense of the material as a whole and gain an understanding of the essential meaning of the text. Third, while keeping the aim clearly in focus, meaning units were identified, that is, sentences or paragraphs that belong together through content or context. Fourth, meaning units were condensed into shorter text, albeit with the core content preserved. Fifth, codes that concisely describe the condensed meaning units were developed, which were then compared and sorted by similarities and differences. Sixth, the first and last author identified, first individually and then together, potential subcategories from the codes and then grouped subcategories with similar content into categories. To remain as faithful to the data as possible, both authors read back and forth between units of meaning, codes, subcategories, and categories throughout the analysis. Moreover, all authors discussed and reflected together at different levels of abstraction and interpretation possibilities [44] until they agreed on the most likely interpretation of the data. The fact that all the co-authors are senior researchers with different skills and perspectives (AS is a professor of informatics and work-integrated learning, SP is a nurse and professor of health sciences and work-integrated learning, IS is a district nurse and associate professor of primary care) gave the analysis process depth and breadth and enhanced reliability [45]. To enhance credibility, each subcategory includes representative quotations from the transcribed text [42]. However, to ensure confidentiality and reduce the possibility of identifying the source of the quotes, the professional role of the informant is excluded, and only their focus group membership is indicated. See examples from the analytical process in Table 2.

**Table 2:** Examples from the analytical process

Meaning units	Condensed	Code	Subcategories	Categories	Theme
“It felt like we were pretty well-prepared for it [the pandemic], because we have had this way of working before. It was nothing strange. We had to rethink completely, but we did, and it worked.”	We were prepared for the pandemic because we were used to this way of working. We had to rethink, and it worked	Prepared to think new	To feel prepared för the unexpected	Benefits of employee-driven innovation	Standing together at the helm
“I think the patients demand it [innovation] too. They demand more and more compared to before…they demand that you keep up.”	The patients demand innovation and that you keep up	Patients expect development of healthcare	Recognising the need for change	Motivating factors for practising employee-driven innovation	
“It took me a while to even understand what we were doing when we got together in here on Tuesdays and yet a very proud manager had told me at my introduction about this [Lean] board and how to use it.”	It took time for me to understand the way of working with innovation even if the manager explained it at my introduction	Takes times to understand employee-driven innovation	Takes time to understand employee-driven innovation and see the benefits	Challenges in practising employee-driven innovation	

## Results

The aim of this study is to explore employees’ experiences of employee-driven innovation in a primary care context. The findings are presented with the overarching theme “Standing together at the helm” followed by three categories: “Motivating factors for practising employee-driven innovation”, “Challenges in practising employee-driven innovation” and “Benefits of employee-driven innovation”, with nine subcategories (Table 3). The overarching theme reflects how employee-driven innovation induced a process of employee empowerment. Employee-driven innovation enabled the employees to learn and jointly change, develop and adapt work practices to ever-evolving circumstances, which can be likened to the helm of a ship. Over time, a collective agency developed, which means that the employees experienced an ability to handle and steer the ship (work practice) together.

**Table 3:** Overarching theme, categories and subcategories

Overarching theme	Standing together at the helm		
Categories	Motivating factors for practising employee-driven innovation	Challenges in practising employee-driven innovation	Benefits of employee-driven innovation
Subcategories	Recognising the need for change Offers opportunities to influence your own workplace Improves local care practice	Takes time to understand employee-driven innovation and see the benefits To persevere in a demanding and task-oriented practice Lack of external support to drive and implement innovative ideas	Improves team spirit and reduces hierarchy To feel prepared for the unexpected Provides a space for learning

### Motivating factors for practising employee-driven innovation

Motivational factors were the very driving force for practising employee-driven innovation. One such factor was to recognise the need for change. Moreover, it was motivating to be able to influence the workplace and improve local care practice.

#### Recognising the need for change

The employees emphasised that innovation is important for several reasons. First, the employee-driven innovation work itself was initiated based on an urgent need for change at the primary care centre. Before it started, the work situation was so strained and challenging that a change was absolutely necessary in order for the employees to want to continue working at the primary care centre. It was not possible to ‘run faster’ but new ways of working were needed to save time and improve the quality of care.

“Sometimes it feels like we were a sinking ship that needed to start floating again, because we couldn’t carry on working the way we were. We had to change something, so we didn’t go down with the ship.”

Focus Group 1

Furthermore, they described that the tough competition in primary care, both between primary care centres and digital health service alternatives, meant that they needed to be at the forefront to survive as a primary care centre in a sparsely populated area. Working with employee-driven innovation was seen as a way to market the primary care centre, to attract both healthcare professionals and patients.

“... we sell services, and we need to market ourselves and make our customers want what we can offer, and we have quite tough competition.”

Focus Group 1

The employees also stressed that healthcare in general needs to adapt to a changing society. New technologies and new societal challenges require innovative ways of working. In addition, the employer, policy makers, stakeholders and well-informed patients and their relatives all expect primary care to keep pace with technological developments.

“The whole society is innovative. You have to keep up!”

Focus Group 3

#### Offers opportunities to influence your own workplace

Being able to develop and improve work practice and, by extension, the work environment, was a major motivating factor for practising employee-driven innovation. The employee-driven innovation intervention was a forum for addressing work-related everyday problems and provided opportunities to discuss and jointly solve problems, which in turn facilitated problem management, reduced frustration, and increased job satisfaction.

“- ... when I started working here, I felt for the first time that someone seemed to listen to what those of us on the floor had to say [...]

-*Why is that important?*

- Eh yeah, that … that you address the issues and do something about them. It becomes more motivating.”

Focus Group 2

The employees felt empowered by taking part in forming and developing the workplace through idea generation and problem solving. This “bottom-up” approach to organisational development felt much more meaningful and humane than getting top-down orders on how to work.

“Here [at this workplace] you can really be involved and have an influence and say what you think and come up with ideas and suggestions, instead of just getting instructions fed from above that you must carry out. […] It is enjoyable to be involved in developing the workplace we work at. Because that is what we do with this.”

Focus Group 2

#### Improves local care practice

The employees experienced that the employee-driven innovation efforts improved care and increased efficiency, which was both inspiring and motivating. The common work routines that were developed clarified the responsibility of different professional roles in the care chain, which reduced the risk of mismanagement that might threaten patient safety and/or prolonged the patient journey. Thus, the routines created a sense of security. Examples of improvements included new ways of handling prescription requests and managing the telephone queue which increased telephone accessibility, an improved triage and developing new working methods to eliminate waiting lists. Altogether, the new work routines facilitated daily practice, improved care and saved time.

“After a while we saw for example how much time we saved by coming up with better ways of working. And time is something we need more of.”

Focus Group 2

The employees’ context-specific knowledge and understanding of local needs was a key element that enabled them to develop new and improved ways of daily working. Thus, the innovations were first and foremost for the local practice.

“This [innovation] work is meant for us, right? [...] That’s what I think...that you start from here. What can be built up and strengthened here?”

Focus Group 1

### Challenges in practising employee-driven innovation

It was challenging for new employees to learn the employee-driven innovation approach and recognise the long-term benefits. Another challenge was to persevere with the innovation work when practice is demanding and task oriented. Also, the employees experienced that they could not proceed with some ideas as they lacked external support.

#### Takes time to understand employee-driven innovation and see the benefits

New employees found it challenging to understand and become comfortable with the way of working with employee-driven innovation. To be involved in developing work practice was a new experience, as in previous workplaces they had been used to getting top-down directives on what and how work should be done. Although they appreciated the innovative way of working, they were not used to prioritising innovative and creative work at the expense of clinical work.

“…[at previous workplaces] you should only do what you are told to, without questioning so much, so I thought it felt very strange that we would spend a whole hour each week doing ‘nothing’ [making quotation marks with fingers], if you know what I mean. Of course, it *is* important, but it felt very strange at first.”

Focus Group 1

The outcome of the innovation work, which mostly consisted of small improvements in working methods, accumulated over time and eventually lead to improved local care practice and a better working environment. The employees who had been involved since the innovation work began pointed out that new employees had a harder time to perceive the benefits of employee-driven innovation, since they had not experienced the improvements that had taken place over time. Thus, the new employees lacked an important perspective.

“Those of us who have been on the whole journey […] have seen the great benefits and how much we have changed. If you come in the middle, it may take a while before you see the big advantages with it [employee-driven innovation]. We’ve been here from when it [the work environment] was very bad and seen it improve. Some may think that everything is fine, it’s all okay. Do we really need to spend so much time on this?”

Focus Group 2

#### To persevere in a demanding and task-oriented practice

Even though all employees agreed that practicing employee-driven innovation was both rewarding and important, it was challenging to prioritise it when the pace of work was high, and they struggled to keep up with their ordinary work tasks. In these situations, the focus was mostly on catching up with the clinical work and the motivation to participate in the innovation work decreased. This became particularly evident when additional tasks were added, such as introducing new staff or vaccination against Covid-19. Therefore, there were contradictory feelings at times.

“… it feels a bit like a ‘waste’ of time for me, who has so much to do […] there is so much [ordinary work] you are behind with all the time […] It’s a bit double-edged. At the same time, it is a bit fun to do something else sometimes […].”

Focus Group 3

Sometimes, when the workload was too high and the employees had fallen behind with their clinical work, the innovation work had to be put on hold briefly. This also applied when the workforce was reduced due to annual leave, for example over the summer.

#### Lack of external support to drive and implement innovative ideas

Although the employees felt that the management within the organisation allowed them to pursue employee-driven innovation, top-down orders sometimes interfered with their new ways of working. Moreover, they lacked external practical support to solve issues that arose during the innovation process, particularly in areas such as IT, which lay outside their area of expertise. Consequently, some improvements could not be implemented which was frustrating.

“... we worked out new ways of working and had a lot of ideas, but I feel that we were stopped from the top ‘no, but that is not possible’, even though we had a lot of good ideas.”

Focus Group 3

However, during the pandemic, video consultations and other technical solutions became possible, which they had previously been told were not possible to implement. Thus, the pandemic drove technological development, which was perceived as something positive.

### Benefits of employee-driven innovation

Practising employee-driven innovation improved the team spirit among the employees. It also fostered a creative mindset that prepared the employees for unexpected situations. Moreover, the innovation work enabled learning by providing opportunities for exchange of knowledge, perspectives, and ideas.

#### Reduces hierarchy and improves team spirit

Before the employee-driven innovation work began, there was a more pronounced hierarchical culture at the primary care centre, where decision-making power was unevenly distributed between the professional groups. By working in interprofessional teams, however, hierarchical structures were dissolved over time. Through the interprofessional collaboration, the employees gained an increased understanding of the challenges and work situation of the various professions, which contributed to increased respect between the professional groups. It also became evident that the skills, competences, and expert knowledge of all professions were needed to find sustainable solutions to the innovation problems. The employees thereby gained an increased understanding that the entire care chain at the primary care centre needs to function in order to provide safe and high-quality care, and thus the knowledge and perspectives of all professions were equally important and valuable in the innovation work. As a result, the employees felt confident to share their ideas with each other, regardless of profession or position.

“…and there is not this hierarchy here that I have come across in many other places, but rather all employees have a value here.”

Focus Group 3

“I admit, when there is a problem, I am the first to come up with a solution because I think I have the absolute best solution to it. I have learned that I certainly do not. [...] I have gained much greater respect for others around me, who do not think or feel the same way I do.”

Focus Group 1

The increased awareness of the equal value of all professions improved the dialogue and collaboration between the different professional groups, in clinical practice too, and enhanced the sense of cohesion. Consequently, the employee-driven innovation efforts built bridges between professional groups and broke down silo thinking. As team spirit increased, potential conflicts between the different professional groups at the primary care centre decreased, and they took joint responsibility for solving emerging work-related challenges and problems.

“It is a lot ... yes, a lot more fun when you are *one* team. When you work together towards one goal instead of people fighting and holding on to their things and ... trying to shift their own work tasks onto another professional group.”

Focus Group 2

#### To feel prepared for the unexpected

Through the employee-driven innovation approach, employees learned to handle work-related challenges and find creative solutions. To be innovative, the employees needed to be open-minded, curious, original, brave, forward-looking, and change-oriented and thereby they developed a progressive and solution-oriented mindset rather than a problem-focused one. In this way, they gained a collective confidence in coping with challenges they faced in their everyday work. Further, this new mindset resulted in action readiness and a sense of being prepared for unforeseen challenges such as a pandemic.

“...when the pandemic came, I found that we were quite equipped to think new, right…and we were all involved ‘Yes, but can we do like this?’ We were spouting new ideas all the time [...] Everyone was involved and really tried: ‘we have to solve this now, what shall we do?’. […]...it felt like we were pretty well-equipped just because we have had this way of working before. It was nothing strange. We really had to rethink, but we did and... it worked!”

Focus Group 2

Moreover, in the spirit of innovation, crises and problems were not perceived primarily as threatening or stressful but, although challenging, rather as assets as they sparked creativity and innovation needed to drive development forward.

“But the pandemic has also contributed to something great, that people had to think outside the box and just find solutions. […] So, I think the pandemic is not only bad.”

Focus Group 1

#### Provides a space for learning

The work at the primary care centre was often demanding, comprising high pace of work, unpredictability, and complex care situations. During the weekly innovation hour, the employees could disconnect from their clinical work for a while to interact with colleagues, share knowledge, experience and perspectives on issues or problems that arose in daily work practice. These ‘spanners in the works’ became the starting point for joint knowledge creation, learning and innovation and thus drove the development of care practice. The employees’ various professional and personal backgrounds enabled new perspectives and learning and were therefore seen as an asset in the innovation work. In this way, the innovation hour became a space that nurtured individual and collective learning.

“-It is good with differences. Then you can see things from different perspectives and hopefully learn from each other.

-You are allowed to be different here too.

-Yeah, that is good!”.

Focus Group 3

The employees also saw the innovation work as an opportunity to interact with other practices in order to learn from each other’s mistakes as well as benefit from each other’s innovations. By avoiding ‘reinventing the wheel’, innovation work can be more efficient. However, they found that, unlike the managers, ‘ordinary’ employees largely lacked organised and recurring forums in which they could interact with healthcare professionals from other primary care centres. Rather, exchanges often took place during such events as occasional study visits. The employees gave an example of how they had improved their triage after a visit to another primary care centre.

“We changed so that we [nurses] sit together with doctors instead, to increase competence. We work in a completely different way than we did before.”

Focus Group 3

## Discussion

This study aims to explore employees’ experiences of employee-driven innovation in a primary care context. The overarching theme of “Standing together at the helm” illustrates the role of employee-driven innovation in the employee empowerment process. Like the helm of a ship, employee-driven innovation enabled the employees to learn, both individually and collectively, and jointly shape, develop and adapt work practice to internal and external demands. Thus, the employees felt equipped to handle unexpected situations and over time, as they saw the benefits of employee-driven innovation (improved care practice, time savings, improved work environment), a collective agency developed. Thus, the employees had both the tool to steer the ship (work practice) and the confidence that they could jointly manage to do so. However, to succeed and endure over time, it requires both a mandate and the provision of allocated time resources.

### Enabling adaptive primary care organisations

This study highlights the enabling role of employee-driven innovation in achieving adaptability in a primary care context. Adaptability can be conceptualised as a creative problem-solving process that alters routines in an organisation in response to internal or external changes [46], such as new opportunities, problems, technologies, ideas and methods. In accordance with earlier research [47, 48] our findings suggest that there is a strong link between learning, innovation, and adaptability. By engaging in innovation, the employees learned problem-solving skills, to collaborate, to develop new working methods, to be prepared for unexpected situations, to be open-minded and aware of colleagues’ skills and competences, both as individuals and as a group. This finding suggests that employee engagement in innovation involves three mutually occurring and synergistic change processes, namely (I) individual learning, (II) collective learning, and (III) transformation of workplace practices. This can be seen as an extension of Billett’s [11] conceptualisation of innovation at work as two interdependently occurring change processes: (I) individual’s learning and (II) the transformation of workplace practices.

Furthermore, by engaging in employee-driven innovation, the employees learned that they jointly could handle, find solutions, and adapt to challenges that arose in everyday working life as well as in crises, which developed a collective agency. This collective problem-solving confidence improved the employees´ adaptive capacity, which in turn formed the basis for a more adaptable organisation that could more easily respond to internal and external challenges. As contemporary workplace practices and work requirements change in response to technological advancements and changing product and service requirements, individuals in all occupations need to continue learning throughout their working life [49]. Fundamentally, it is about individuals learning to adapt what they know to other circumstances and new challenges and thus contribute to individual, collective and organisational learning.

### Employee-driven innovation—a challenge in task-intensive work practices

Our findings show that the employee-driven innovation efforts at the primary care centre resulted in new common work routines that standardised workflows, which according to the employees, in the long run, saved time, improved local care practice and increased job satisfaction. This aligns with prior research highlighting how incremental, practice-driven change processes can ultimately transform care systems and enhance care practice [50–52]. However, while the employees in this study were predominantly positive towards the innovation efforts, a certain ambivalence could still be discerned. Although they recognised the necessity to improve and develop practice, there was also a sense of stress associated with devoting time to these efforts amidst many other pressing tasks. This was particularly evident in new employees, who were not used to engaging in employee-driven innovation and had not yet seen the long-term benefits. In a time-pressured and harsh work environment, it is easy to get caught in what Chesluk and Holmboe [53] refer to as the ‘frantic bubble’, where employees try to cope with the overwhelming workload by working faster and harder. When faced with significant time pressure challenges and the urgency to address immediate issues, there is a tendency to revert to familiar coping mechanisms to manage the workload. To mitigate this risk, clear strategies must be in place to sustain employee-driven innovation efforts over time. One such coping strategy employed was to temporarily suspend innovation efforts for a predetermined period, which requires open and trusting communication between the employees and the management. The importance of trust-based and supportive management to drive and continuously sustain employee-driven innovation efforts has been highlighted in several studies [50, 54–57].

### The importance of organisational support

To reach its full potential, the employee-driven efforts needed support from other departments within the organisation, such as IT. However, the absence of practical support resulted not only in anticipated improvements failing to materialise, but also in employees growing frustrated and demotivated due to a perceived lack of organisational support in their endeavours. Similar to other studies [57, 58], our result reveals a tension between high commitment to perceived needs in clinical practice and other organisational demands, such as rigid rules, prioritisation of resources and assignments. Organisational silo structuring and constraining conditions within the organisation hamper employee engagement to drive innovation [58]. This underscores the import﻿﻿ance of instilling the spirit of employee-driven innovation throughout all levels of the organisation, emphasising the necessity for support functions to possess both resources and willingness to bolster employee-driven innovation efforts at local primary care centres.

### Arranged space for learning and collaboration to drive innovation

From a sociocultural perspective, the weekly innovation hour served as an arranged space for learning in which the interprofessional teamwork was a key element. Enabling learning environments require, amongst other things, both formal and informal (e.g. tea or coffee breaks, lunches) arenas for planning and knowledge exchange [59, 60]. Accordingly, our findings emphasise the need to integrate formal arenas for learning and innovation into practice, as the task-intensive and production-oriented environment of primary care rarely allows room for improvised activities that can drive innovation and development of local care practice. This arranged learning space formed the very basis for what Lemmety and Billett [32] coin as employee-driven learning and innovation (EDLI). In the problem-solving activities, each profession contributed their unique knowledge, expertise, and perspective to the innovation process, ensuring that the new work routines applied to all professional groups at the primary care centre. As outlined in the theory of ZPD [26], the teamwork and collaborative efforts expanded the employees’ understanding beyond their individual professional specialties, fostering a comprehensive and inclusive approach to healthcare delivery. Although the theory of ZPD originally suggested that there must be an intellectual asymmetry between collaborating peers for effective learning, our findings support more recent research that indicates that learning also occurs when individuals with similar levels of conceptual understanding collaborate, including collaboration between experts (see [61]) or students (see [62]).

An increasing body of research indicates that merging Lean practices (typically centred on waste elimination) with innovation (which inherently involves accepting risk and potential waste to pursue genuinely new ideas) can pose both challenges as well as synergies [63–65]. In this study, the Lean approach encompassed the philosophy of continuous improvement and waste reduction, augmented by the use of the Lean board tool. We found that the Lean board served as structure for the innovation process and thus facilitated to frame and render the abstract concept of innovation comprehensible for the employees. Thus, from a sociocultural perspective the Lean board functioned as an example of a culturally mediated artefact [27] that structured and fostered communication, collaboration, and goal alignment in the employee-driven innovation processes. Nonetheless, the way of structuring and organising innovation efforts requires continual evaluation and refinement as the work evolves over time.

### Strengths and limitations

This study contributes with significant knowledge about what motivates employees in the primary care context to engage in employee-driven innovation work, as well as what opportunities and challenges exist. Furthermore, it increases our understanding on how employee-driven innovation in a primary care context may enhance collective agency and individual and organisational adaptability. The results are useful for managers, both at the primary care centre level and higher up in the organisation, in their endeavours to develop care by promoting and utilising the employees’ competences and innovative abilities.

The study has some limitations. First, it was conducted in a rural primary care centre which for several years suffered from high workload and difficulties in recruiting and retaining staff. These challenging circumstances may have influenced experiences of employee-driven innovation. However, these challenges are common in primary care, albeit to varying degrees, which indicates that the study’s results may be transferred to other primary care contexts. Despite this, we suggest that future research on employees’ experiences of employee-driven innovation includes studies that cover a variety of primary care centres (large/small, urban/rural) to ensure transferability.

Second, as described earlier, this study explores second-order employee-driven innovation which involves both bottom-up and top-down processes. Nevertheless, we cannot overlook the fact that the experiences will be different if employee-driven innovation is organised differently, as a pure bottom-up (first-order) or a more top-down (third-order) process. 

Third and finally, as the first author works as a district nurse at the present primary care centre and is engaged in the employee-driven innovation work, this study was conducted from an insider research perspective. Researchers who are involved in their research context risk not being sufficiently objective in their research, but let personal values and experiences influence the research process [20]. To minimise bias, the first author endeavoured to continually reflect on how her pre-understanding and her dual role as a district nurse and researcher might influence the research process. This was also frequently discussed with co-authors. Furthermore, given the first author’s rich involvement in the innovation efforts at the primary care centre, she might have been perceived as favourably disposed regarding the ongoing innovation efforts. This might have hampered participants from fully reflecting on all facets of their experience [66], potentially neglecting the more negative aspects. Also, organising the focus groups as interprofessional sessions rather than separate groups for each profession may have prevented participants from addressing more sensitive issues, such as potential inherent conflicts and competition between professional groups. Consequently, the findings may not capture the full complexity of the phenomenon. However, qualitative research hinges on the establishment of trust between researchers and informants, fostering an environment where informants willingly contribute their knowledge with a shared commitment to generating pertinent insights [67]. We therefore endeavoured to promote a sense of security, explicitly encouraging all participants to share both positive and negative experiences, emphasising that all experiences were equally valuable. To enhance the trustworthiness of the results, member-checking was employed [68]. Three employees (one nurse, one medical secretary and one resident physician) were asked to read, comment, correct or clarify the result as appropriate with their experiences. Additionally, the findings and potential ethical concerns were shared and discussed with all employees during a workplace meeting. The member checking process did not lead to any changes, but the results were confirmed by the employees.

## Conclusions and future research

This study contributes with knowledge about how employees in primary care experience employee-driven innovation in terms of motivation, challenges, and benefits. This knowledge can support the management at different organisational levels in primary care in their efforts to promote innovation and development. The result can also support the employees’ empowerment process and contribute to developing a collective agency which in turn can generate an ability to adapt, both on a personal and organisational level. In this way, employees can learn, both individually and collectively, and jointly shape, develop and adapt work practice to internal and external requirements. This study also reveals several challenges in sustaining employee-driven innovation efforts within primary care contexts. The high workload and task-intensive environment, commonly found in primary care settings, often diminish employees’ motivation to participate in innovation, particularly among new employees who have yet to experience the long-term incremental improvements resulting from employee-driven initiatives. In addition, organisational silo structures prevent employee-driven innovations from gaining the support needed to be implemented in clinical practice. Given that the findings of this study are based on a single primary care centre, we propose further research to explore how employee-driven innovation influences organisational adaptability within the primary care context. Additionally, there is a need for additional empirical evidence on integrating sustainable employee-driven innovation and learning environments into clinical practice in primary care settings. Furthermore, an intriguing yet under-researched area is understanding the synergies and conflicts that emerge when Lean principles and employee-driven innovation converge in healthcare settings.

### Supplementary Information

Supplementary Material 1.

## Data Availability

The datasets used and/or analysed during the current study are available from the corresponding author on reasonable request.
