# Supplementary material for: Standing together at the helm – how employees experience employee-driven innovation in primary care
Source: BMC Health Serv Res. 2024 May 22;24:655. doi: 10.1186/s12913-024-11090-0 (PMC11110197; doi:10.1186/s12913-024-11090-0)
Supplement: Supplementary file 1 — Supplementary Material 1. [file 12913_2024_11090_MOESM1_ESM.docx]

**Focus Group Interview Guide**

1. Could you please describe how the journey of innovation began at the healthcare center?

2. What does innovation and innovation work mean to you? Please describe and share your thoughts.

- How would you define innovation?

3. In what ways do you engage in innovation? Share your methods, elaborate on them, and provide examples.

- How do you personally contribute to innovation at the healthcare center?
- What role does the manager play in the process of innovation?
- What is the staff's role?
- How important is reflection for the innovation process?

3. Is it essential to invest in innovation work? If yes, why? Please elaborate.

- Is it a significant priority? For whom?
- Are there incentives for participating in innovation work? If so, could you specify what they entail?

4. Are there expectations for you to engage in innovation work?

- If so, where do these expectations originate from? (e.g., the employer/organization, healthcare center manager, colleagues, patients)
- Who bears the responsibility for driving innovation efforts?

5. What resources are necessary to integrate innovation as a fundamental part of your daily work routine?

- What resources are required, both within the healthcare center and externally?
- What type of support is needed, both within the healthcare center and externally?

6. What opportunities, challenges, or obstacles do you encounter when adopting your current approach to innovation?

7. Could you describe your experiences in working with innovation as part of your daily responsibilities?

- How do you balance demands and available resources?
- What advantages or disadvantages do you observe, whether on a personal, professional, organisational, work environment, or patient-related level?
- How do you feel when an idea or proposal fails to yield the desired results?
- Does innovation work have an impact on the work environment, and if so, in what ways?

8. Is there an alternative approach to innovation work that you believe might be more effective than your current methods?

9. Of all the topics we've discussed today, what stands out as the most significant?

Formulärets överkant
